# Genome-Wide Generation of Yeast Gene Deletion Strains

**DOI:** 10.1002/cfg.95

**Published:** 2001-08

**Authors:** Diane E. Kelly, David C. Lamb, Steven L. Kelly

**Affiliations:** Institute of Biological Sciences University of Wales Aberystwyth Aberystwyth, Wales SY23 3DA UK

## Abstract

In the year 2001 a collection of yeast strains will be completed that are deleted in the 6000 open reading frames selected as putative genes by the initial bioinformatic analysis of the *Saccharomyces cerevisiae* genome. The collection was produced by the transatlantic yeast gene deletion project, a collaboration involving researchers in the USA, Canada and Europe. The European effort was part of EUROFAN (European Functional Analysis Network) where some of the strains could feed into various functional analysis nodes dealing with specific areas of cell biology. With approximately 40% of human genes involved in heritable disease having a homologue in yeast and with the use of yeast in various drug discovery strategies, not least due to the dramatic increase in fungal infections, these strains will be valuable in trans-genomic studies and in specialised interest studies in individual laboratories. A detailed analysis of the project by the consortium is in preparation, here we discuss the yeast strains, reported findings and approaches to using this resource.


**Comparative and Functional Genomics**
*Comp Funct Genom* 2001; **2**: 236–242.
**DOI:** 10.1002/cfg.95

Review

### Genome-wide generation of yeast gene deletion strains

Diane E. Kelly*, David C. Lamb and Steven L. Kelly

*Institute of Biological Sciences, University of Wales Aberystwyth, Aberystwyth, Wales, UK SY23 3DA*

**Correspondence to:*
*D. E. Kelly, Institute of Biological Sciences, University of Wales Aberystwyth, Aberystwyth, Wales SY23 3DA, UK.*
*E-mail: dek@aber.ac.uk*

Received: 26 June 2001
Accepted: 27 June 2001

### Abstract

**In the year 2001 a collection of yeast strains will be completed that are deleted in the 6000 open reading frames selected as putative genes by the initial bioinformatic analysis of the *Saccharomyces cerevisiae* genome. The collection was produced by the transatlantic yeast gene deletion project, a collaboration involving researchers in the USA, Canada and Europe. The European effort was part of EUROFAN (European Functional Analysis Network) where some of the strains could feed into various functional analysis nodes dealing with specific areas of cell biology. With approximately 40% of human genes involved in heritable disease having a homologue in yeast and with the use of yeast in various drug discovery strategies, not least due to the dramatic increase in fungal infections, these strains will be valuable in trans-genomic studies and in specialised interest studies in individual laboratories. A detailed analysis of the project by the consortium is in preparation, here we discuss the yeast strains, reported findings and approaches to using this resource. Copyright © 2001 John Wiley & Sons, Ltd.**

### Introduction

The budding yeast *Saccharomyces cerevisiae* serves as a central model eukaryotic organism in biological research and is important in biotechnology. Completion of the *S. cerevisiae* sequencing project, the first for a eukaryotic genome, indicated the presence of approximately 6000 putative open reading frames (ORFs) that are likely to encode protein products in the yeast cell (Goffeau *et al.*, 1996). Initial analysis of the sequence revealed the existence of approximately 6275 ORFs that theoretically could encode proteins greater than 99 amino acids and attempts were made to classify yeast proteins according to their predicted function. Initially it was estimated that the yeast cell devotes 11% of its proteome to metabolism; 3% to energy production and storage; 3% to DNA replication, repair and recombination; 7% to transcription and 6% to translation. Some 430 proteins were predicted to be involved in protein targeting or intracellular trafficking, 250 as structural proteins, 200 as transcription factors and 250 involved in transport. However, approximately 50% of ORFs contained within the yeast genome were of no known function.

A long-standing approach for the determination of gene function has been through the phenotypic analysis of mutants missing the particular gene of interest, but many genes were not identified by classical genetic approaches. The specific deletion of a yeast gene in a directed approach involves generation of a deletion cassette by the polymerase chain reaction (PCR) (Wach *et al.*, 1994) and takes advantage of the high level of homologous recombination in yeast. Short-flanking homology of only 40 base pairs allows a precise replacement of an open-reading frame with a selectable marker. Conceptually, as well as practically it became possible to envisage deleting each of the ORFs identified by the yeast genome sequencing project and this was the strategy adopted by a consortium of yeast laboratories based both in Europe and North America. Within the deletion cassette produced for each ORF modifications were introduced that can mark the specific gene with a unique identifier (or molecular barcode) so that serial analysis of phenotypes of mutant strains becomes easier.

### European functional analysis network (EUROFAN)

The first EUROFAN programme established an approach whereby the function of a particular gene

Copyright © 2001 John Wiley & Sons, Ltd.

could be determined with ever increasing specificity, by moving it from one level of analysis to another. This hierarchical scheme was divided between different participating groups, or Nodes, that concentrated on a particular aspect of cell structure or metabolism (Table 1). This organisation was efficient since communication between groups meant that not all genes had to have every analysis performed on them. It is pertinent that this systematic functional analysis was not intended to replace "normal" biological research, but it was a stated aim that analysis of each novel gene could be taken to a particular level whereby an interested laboratory could incorporate it into its own research programme.

The first level of analysis was the so-called B0 phase, where deletion strains and replacement cassettes were generated for ORFs in the *S. cerevisiae* genome. Under EUROFAN's first programme a thousand open-reading frames were targeted for deletion using a similar strategy to the later transatlantic project, but where the molecular barcodes were absent. These were undertaken in Europe through the distribution of sets of six genes (so-called 'six packs') to various laboratories.

### Transatlantic yeast gene deletion project

In the second EUROFAN programme, the B0 node was part of the transatlantic consortium undertaking the task of generating a genome-wide set of gene deletion strains beginning in 1998. The participants in the project are indicated in Table 2. A total of 6138 ORFs were subjected to targeted deletions in a diploid reference strain, BY4743 (Table 3). ORFs were organised into groups of 96 starting from the left arm of a chromosome, so that participating laboratories were assigned sets of primers distributed in a multi-well format to generate PCR products for targeted deletion, with further sets of confirmation primers also in the same format. A major challenge to the success of the project was the design and synthesis of primers targeted against such a large number of ORFs. All the primers used in this work were designed by computer algorithms and synthesis co-ordinated at the Stanford Resource Centre. Primer sequences and ORF locations were chosen from the Stanford Genome Database (**http://genome.www.stanford.edu/Saccharomyces/**). Although ~10% of the predicted ORFs in *S. cerevisiae* overlap one another, the positions of the deletions were not adjusted and no

Table 1. Hierarchy of Eurofan consortia. Level A: service or material resources for the whole of Eurofan. Level B: provided the first level of searching for function and permitted approximate assignments that allowed individual genes to be passed to the next more specific level. The functional analysis nodes were divided into three clusters, F1, F2 and F3

| | | | |
|---|---|---|---|
| | **Eurofan Resource Consortia** | | |
| A1 | Central coordination | | |
| A2 | Informatics | | |
| A3 | Industrial Liason/Yeast Iindustrial Platform (YIP) | | |
| A4 | Genetic Archive & Stock centre | | |
| | **Basic Fundemental Analyses** | | |
| B0 | High throughput generation of deletants | | |
| B1 | Qualitative phenotypic analysis | | |
| B2 | Transcript analysis | | |
| B3 | Essential genes | | |
| B4 | Integrative approaches to to functional analysis | | |
| | **Function Analysis Nodes** | | |
| F1 | Nuclear dynamics | N1 | Genome Plasticity/Nuclear Structure/Meiosis |
| | | N2 | Chromosome Structure/Architecture & Biology |
| F2 | Metabolic interactions | N3 | Transport |
| | | N4 | Organelles |
| F3 | Cell Organisation & Development | N5 | Secretion & Protein Trafficking |
| | | N6 | Cell Architecture |
| | | N7 | Cell Walls & Morphogenesis |
| | | N8 | Development |

Copyright © 2001 John Wiley & Sons, Ltd. 

Table 2. Members of the Transatlantic Yeast Gene Deletion Consortium

| Consortium Member |
|---|
| **U.S./Canada** |
| Acacia Biosciences, Richmond, California |
| Jef Boeke, Johns Hopkins University |
| Howard Bussey, McGill University |
| Ron Davis, Stanford University |
| Mark Johnston, Washington University at St. Louis |
| Jasper Rine, University of California at Berkeley |
| Rosetta Inpharmatics, Kirkland, Washington |
| Jeffery Strathern/David Garfinkel, Frederick Cancer Research and Development Center |
| Michael Snyder, Yale University |
| **EUROFAN:** |
| Bruno Andre, University Libre de Bruxelles |
| Francoise Foury, Universite Catholique de Louvain |
| Johannes Hegemann, Justus-Liebig-Universitaet Giessen |
| Steven Kelly, University of Wales Aberystwyth |
| Peter Philippsen, Biozentrum, Basel |
| Bart Scherens/ F. Messenguy, Institut de Recherches du CERIA |
| Jose Revuelta, Universidad de Salamanca |
| Giorgio Valle, University of Padova |
| Guido Volckaert, Katholieke Universiteit Leuven |

attempt was made to avoid known essential genes, genes in which a previous deletion had been constructed, or genes with a well defined function. Genes represented multiple times in the genome were not deleted as their targeted disruption was considered to be problematic.

For the generation and confirmation of each deletion cassette, eight different primers were synthesised for each ORF. Four oligonucleotides (two 74mers and two 45mers) were used in the synthesis of the deletion module used in the yeast transformation. The strategy is given in Figure 1. Specifically, short regions of yeast DNA sequence identical to those found upstream and downstream of a targeted gene are placed at each end of the antibiotic resistance cassette, KanMX4 (U1, D1)

Table 3. Four yeast strains employed in the transatlantic yeast gene deletion project

| Yeast Strains | |
|---|---|
| BY4741 | MATa ura3Δ, leu2Δ, his3Δ, met15 Δ, LYS2 |
| BY4742 | MATα ura3Δ, leu2, his3Δ, MET15, lys2Δ |
| BY4743 | MATa/α ura3Δ/ura3 Δ, leu2 Δ/leu2Δ, his3Δ/his3Δ, met15Δ/MET15, LYS2/lys2Δ |
| BY4743HO | Homozygous diploid gene deletion strain |

though PCR. In addition, two molecular barcodes (UPTAG and DOWNTAG) are introduced into the deletion strain, a 20 bp tag priming site and 18 bases of sequence complementary to the ORF. The deletion cassettes were designed to remove the entire coding sequence for a given ORF, but to leave the start and stop codon intact. These 74mers were used to amplify the heterologous KanMX4 module, which contains a constitutive efficient promoter from a related yeast strain *Ashbya gosspii* fused to the kanamycin resistance gene *nptI*. A second round of PCR using primers with 45 bases of homology to the region upstream and downstream of a specific targeted ORF further improved targeting. Yeast transformations with the PCR product allowed for specific replacement of the targeted gene and gene- deleted strains were selected for by growth on complete media containing the antibiotic Geneticin.

To verify correct homologous recombination of the deletion cassette with the yeast genome, genomic DNA was isolated from the Geneticin resistant colonies and used as template in PCR reactions using two primers common to the KanMX4 module:

Kan B: 5′-CTGCAGCGAGGAGCCGTAAT-3′

Kan C: 5′-TGATTTTGATGACGAGCGTAAT-3′

Four further primers were used to confirm the correct integration of the deletion module into the yeast genome (Figure 2), A, B, C, and D. Primers A and D were from regions up to 400 bases upstream and downstream of the start and stop codons respectively. Primers B and C were from within the ORF. For verification, both the A-KanB and the D-KanC PCR reactions were required to give the correct size product when analysed by agarose gel electrophoresis. If one of the A-Kan B or D-KanC reactions failed to yield a correctly sized product the identification of the correctly sized A-D product sufficed. In addition, haploid deletion strains were tested for the disappearance of the wild type AB and CD products.

For approximately 5000 ORF's the first transformation gave the correct heterozygous deletions as confirmed by analytical PCR. The remaining ORF's have been subject to the same PCR approach using either the original primer sets or using newly designed primers. To date 5935 heterozygous strains are now available as successfully targeted deletions. Of the remaining 'difficult' ORF's, sequence error, aberrant confirmation PCR analysis or unknown factors may account for their not being generated to date.

For each ORF deletion strain viable in a haploid

Copyright © 2001 John Wiley & Sons, Ltd. 

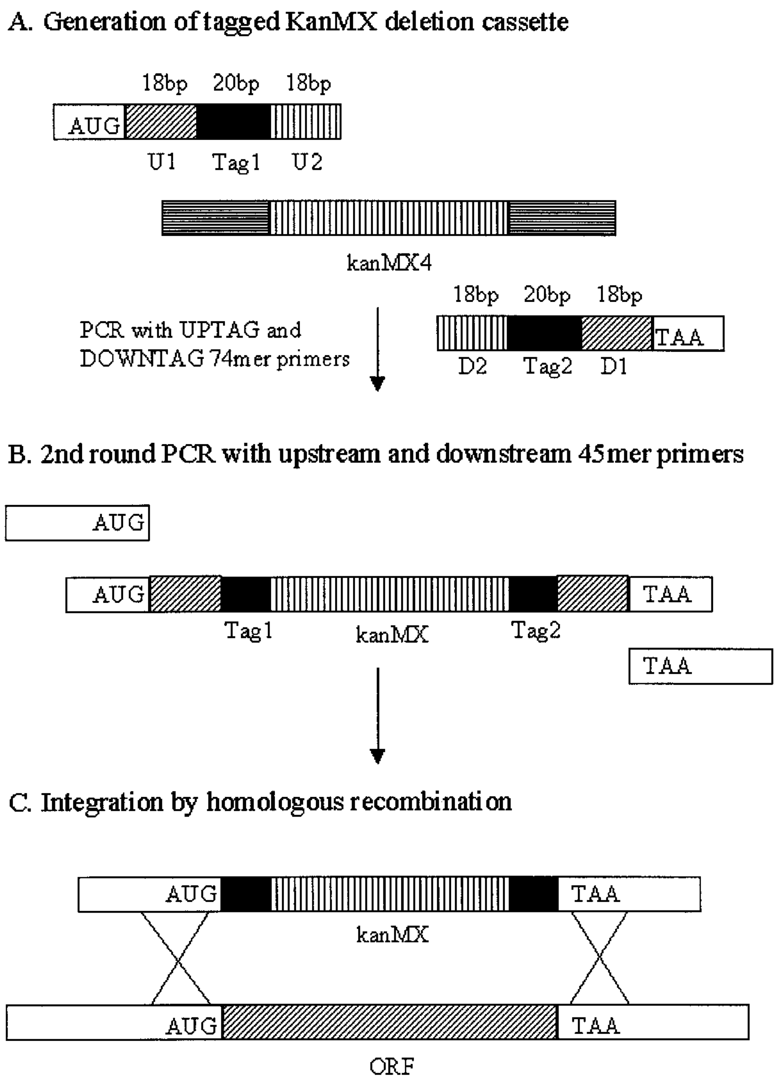

**Figure 1.** PCR strategy for targeted deletion of yeast ORFs. A) First round PCR attaches UPTAG and DOWNTAG sequences. B) Second round PCR extends the sequence to include 45bp homologous to gene to be targeted. C) Following transformation the kanR module with sequence tags recombines at chromosomal locus of targeted gene condition, two haploid strains, one for each mating type, were also generated mainly by tetrad dissection of independent heterozygous deletions in BY4743 and to a lesser extent by direct transformation of haploid strains (Table 2). Four strain types were generated when possible; a diploid heterozygous deletion strain, haploid deletion strains with either mating type corresponding to the genotypes of strains BY4171 and BY4172 and a diploid homozygous deletion strain generated where possible by mating the corresponding two haploid strains from independent deletion events.

Strains obtained in the project were examined for budding patterns, the ability to sporulate, the inability to mate and for slow growth, besides the test for their essential nature as reflected by tetrad analysis. Electronic records were kept for every strain constructed. *MATa* haploid strains were given record numbers of less than 10 000, *MATα* haploid strains were given record numbers between 10 000 and 20 000, the heterozygous diploid, between 20 000 and 30 000 and the homozygous diploid greater than 30 000. Each record is shown to consist of primer sequence information, the

Copyright © 2001 John Wiley & Sons, Ltd. 

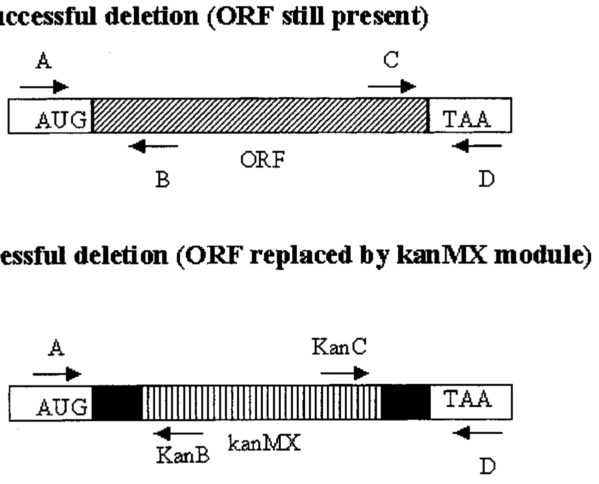

**Figure 2.** PCR strategy to confirm gene deletion. 1) Primers A and D when used with primers B and C respectively were designed to give PCR products in the range of 300–1000 bases. 2) Primers A and D used with primers KanB and KanC respectively confirmed successful targeted deletion event results of the different diploid tests that were performed and notes about the phenotype. Data for all the completed strains can be found at **http://www-sequence.stanford.edu/group/deletion/index.html**. All the generated strains can be obtained from Research Genetics (Huntsville, AL, USA; **http://www.resgen.com**) or EUROSCARF (Frankfurt, Germany; **http://www.uni-frankfurt.de/fb15/mikro/euroscarf/**). They are also available from the American Type Culture Collection. As an additional task the members of B0 in EUROFAN II also cloned all the gene deletion cassettes for their assigned ORFs, which allows their use in other strains of yeast.

### The value and application of a genome set of gene deletants

With the availability of a set of deletants covering almost all the predicted open reading frames of yeast, new opportunities are arising concerning both fundamental and applied studies in comparative and functional genomics. Some deletants may be in open-reading frames that are not genes, some real genes are not currently represented due to the criteria used in selecting ORFs for deletion and a limited number were in open reading frames that were specifically excluded. A re-evaluation of the initial annotation of yeast ORFs has appeared recently, assigning some as spurious (368) and others as very hypothetical (192) [14]. Thus some of the deletions are probably not in real genes, but may have phenotypes if expression of a real gene is altered. The number of new putative genes detected (three) will be easily managed in a further set of gene deletions. Some initial methodology developed during EUROFAN has been applied to gene deletions produced in the EUROFAN I Programme and in the subsequent Transatlantic Yeast Gene Deletion Consortium where the EU partners were within EUROFAN II. Unfortunately, while EUROFAN enabled European laboratories to contribute to the generation of the genome-wide set of deletion strains with the US and Canadian partners, EUROFAN ended at the time the strains became available for genome-wide studies. Consequently the techniques and networks established could not proceed to extensive genome-wide studies under the same umbrella, but nevertheless the strains can now be used for genomic studies in the different areas of interest. The detailed report of the genome-wide gene deletion study is currently being prepared for publication and our discussion is consequently restricted to the implications of the work and how various studies have indicated the way ahead using sets of gene deletion strains comprising a part of the total.

### Lethals/non-lethal deletants and other phenotypes

The early stages of the project produced some of the most readily obtained deletants and allowed an

Copyright © 2001 John Wiley & Sons, Ltd.

intermediate stage report on deletion of 2026 open-reading frames [13]. Of these the most obvious interest lay in the proportion of lethal genes that represented 17% of the total investigated and which were distributed widely. Phenotypes were also studied and here the barcode system, identifying each deletant, could be used to estimate the relative growth of each of 558 viable homozygous deletion strains by hybridisation signal. In this way results were taken and found to tally with expectation. Mutants deleted in genes needed for growth on minimal medium were observed to show reduced growth. The availability of the barcoded deletion strains allowed integrated studies with transcriptome analysis. Interestingly, little up-regulation of genes needed for growth on minimal medium was observed, indicating the value of the possibility of experiments with deletants as a complementary technique to transcriptome studies in pinpointing function. Among all the strains, about 40% showed growth defects.

Further reports have been made into sets of deletants within the project. For instance, Nierdenthal *et al.*, [10] found that among 265 genes on chromosome VIII, 18% of deletants showed a growth phenotype and 18% resulted in lethality with growth on complete medium. They went on to examine other genetic features, while Lucau-Danila *et al.*, [8] attempted deletion of 480 genes (five sets of 96 genes) across chromosomes III, IV, VII, XII, XIV and XV, obtaining 456 heterozygotes and 385 homozygotes. In this study twenty-five new lethal deletions and thirty causing slow growth were identified.

The approach adopted in EUROFAN involved the development of studies within special interest nodes. For instance, approaches to lipid metabolism [1] or cell wall synthesis [2] were undertaken. Reports on the findings of all the function nodes are available at: **http://www.mips.biochem.mpg.de/**.

Identifying and classifying genes involved in cell wall formation was undertaken with a set of deletants produced in EUROFAN I [2], strains without the barcode approach incorporated into the genome-wide deletions undertaken later. In this study 620 strains carrying non-essential gene deletions were screened using selections designed to allow detection of cell wall related phenotypes and 145 showed a phenotype. Extrapolation of their results suggests that almost 1200 yeast genes may cause changes in cell wall production. The screening involved altered sensitivity of strains to calcofluor white, SDS and sonication or else detection of different morphology. Similar simple phenotypic changes detectable by replica plating were favoured in other nodes, such as the lipid node. There, altered sensitivity to osmotic stress or membrane interacting agents such as the antifungal drug amphotericin B were used [1]. Similar approaches for sensitivity to DNA damage were also used. The opportunity to apply these techniques in full genomic studies is now feasible.

### Antifungal and inhibitor mode of action – haploinsufficiency

Almost twenty years ago, the idea of using increased gene copy number to identify the mode of action of inhibitors was demonstrated [11]. In that approach the multi-copy episomal vectors of yeast allow individual transformants containing a plasmid expressing the target to be selected from a library of transformants as a resistant cell line. In the strains generated in the deletion project a new technique was developed and evaluated for revealing haploinsufficiency of drug sensitivity in heterozygous diploid strains [3]. This was based on the increased sensitivity observed for heterozygotes carrying a deletion of the gene encoding the target protein of a chemical inhibitor when compared to strains carrying two wild-type alleles. The increased sensitivity of that strain identifies the gene product as the target of the inhibitor and can be detected rapidly using the unique barcode in hybridisation studies. The proof of principle was established in detail for tunicamycin, an inhibitor of glycosylation, using a pool of 233 heterozygous gene deletion strains. Genome wide studies must now follow these preliminary findings and will reveal full detail on the modes of action of bioactive compounds.

Further studies on the gene deletion strains have integrated the transcriptome approach. Despite the widespread aneuploidy that has been detected in up to 8% of the deletion strains [5,6] useful information has and will be gained. In examining the transcriptome response to numerous bioactive inhibitors, novel modes of action and novel gene functions have been uncovered [5,6]. Many antifungal drugs target ergosterol [7] and transcriptome profiling had pinpointed many novel ORFs with altered transcription in response to such inhibitors. A compendium of responses to different chemical challenges was catalogued and was related to effects of specific gene deletions. A compound with a previously

Copyright © 2001 John Wiley & Sons, Ltd.

unknown mode of action, dyclonine, showed a response indicating sterol biosynthesis inhibition. Specifically, sterol C8-isomerase was the target. This protein and the related sigma receptors of mammals are related in structure and inhibition of C8-isomerase by neuroactive compounds, such as emopamil, as has been observed before [9]. Eight previously orphan ORFs were assigned a function in this study, including proteins required for sterol metabolism, cell-wall function, mitochondrial respiration and protein synthesis. For sterol biosynthesis an unsuspected function in sterol C4-demethylation was revealed. This process requires three activities, an oxygenase, a decarboxylase and a keto-reductase, together with associated electron donors, all of which were previously known genes. The new protein Erg28p may function as a molecular tether for this endoplasmic reticulum associated complex. As sterol C4-demethylation is a long-standing target for antifungal development, this new information is important for designing new strategies for drug discovery.

### Concluding comments

We are entering the 21st century, which will be an era of pre-eminent importance for biology and post-genomics. Yeast has, and will in the future, provide an important platform for fundamental studies on gene functions conserved in plants and man. The genes required for viability in yeast, but absent in man, are good potential targets for antifungals and those essential in yeast and found in man are important for understanding human health and disease. The emergence of fungal infections as a serious medical problem also stimulates the need for such yeast studies in improving the rate of drug development. The availability of a genome-wide set of deletants as a resource to the wider scientific community will lead to further stimulus in the use and value of yeast studies. These will help in the context of big and small science as the gene deletion strains will make yeast studies more accessible to non-specialist laboratories. Yeast studies have again led the way in their approach in the post-genomic era.

### Acknowledgements

We are grateful to the EU for support of the deletion project and BBSRC and MRC for project support.

### References

1. Daum G, Tuller G, Nemec T, *et al.* 1999. Systematic analysis of yeast strains with possible defects in lipid metabolism. *Yeast* **15**: 601–614.
2. De Groot PWJ, Ruiz C, Vazquez de Aldana CR, *et al.* 2001. A genomic approach for the identification and classification of genes involved in cell wall formation and its regulation in *Saccharomyces cerevisiae*. *Comp Funct Genom.* **2**: 124–142.
3. Giaever G, Shoemaker DD, Jones TW, Liang H, Winzeler EA, Astromoff A, Davis RW. 1999. Genomic profiling of drug sensitivities via induced haploinsufficiency. *Nat Genet* **21**: 278–283.
4. Goffeau A, Barrell BG, Bussey H, *et al.* 1996. Life with 6000 genes. *Science* **274**: 546–567.
5. Hughes TR, Marton MJ, Jones AR, *et al.* 2000. Functional discovery via a compendium of expression profiles. *Cell* **102**: 109–126.
6. Hughes TR, Roberts CJ, Dai H, *et al.* 2000. Widespread aneuploidy revealed by DNA microarray expression profiling. *Nat Genet* **25**: 333–337.
7. Lamb DC, Kelly DE, Kelly SL. 1999. Molecular aspects of azole antifungal agents and resistance. *Drug Res Updates* **2**: 390–402.
8. Lucau-Danila A, Wysocki R, Roganti T, Foury F. 2000. Systematic disruption of 456 ORFs in the yeast *Saccharomyces cerevisiae*. *Yeast* **16**: 547–552.
9. Moebius FF, Soellner KEM, Fiechtner B, Huck CW, Bonn G, Glossmann H. 1999. Histidine (77), glutamic acid (81), glutamic acxid (123), threonine (126), asparagine (194) and tryptophan (197) of the human emopamil binding protein are required for *in vivo* sterol $\Delta^{8\rightarrow7}$ isomerisation. *Biochemistry* **38**: 1119–1127.
10. Niedenthal R, Riles L, Guldener U, Klein S, Johnston M, Hegemann JH. 1999. Systematic analysis of *Saccharomyces cerevisiae* chromosome VIII genes. *Yeast* **15**: 1775–1796.
11. Rine J, Hansen W, Hardeman E, Davis RW. 1983. Targeted selection of recombinant clones through gene dosage effects. *Proc Nat Acad Sci U S A* **80**: 6750–6754.
12. Wach A, Brachat A, Pohlmann R, Philippsen P. 1994. New heterologous modules for classical or PCR-based gene disruptions in *Saccharomyces cerevisiae*. *Yeast* **10**: 1793–1808.
13. Winzeler EA, Shoemaker DD, Astromoff A, *et al.* 1999. Functional characterisation of the *S. cerevisiae* genome by gene deletion and parallel analysis. *Science* **285**: 901–906.
14. Wood V, Rutherford KM, Ivens A, Rajandream M-A, Barrell B. 2001. A re-annotation of the *Saccharomyces cerevisiae* genome. *Comp Funct Genom* **2**: 143–154.

Copyright © 2001 John Wiley & Sons, Ltd.
